# Patterns and Perceptions of Standard Order Set Use Among Physicians Working Within a Multihospital System: Mixed Methods Study

**DOI:** 10.2196/54022

**Published:** 2024-11-08

**Authors:** Sundresan Naicker, Amina Tariq, Raelene Donovan, Honor Magon, Nicole White, Joshua Simmons, Steven M McPhail

**Affiliations:** 1 Australian Centre for Health Services Innovation and Centre for Healthcare Transformation School of Public Health and Social Work, Faculty of Health Queensland University of Technology Kelvin Grove Australia; 2 Digital Health and Informatics Directorate Metro South Health Woolloongabba Australia

**Keywords:** medical informatics, adoption and implementation, behavior, health systems, testing, electronic medical records, behavioral model, quantitative data, semistructured interview, clinical practice, user preference, user, user experience

## Abstract

**Background:**

Electronic standard order sets automate the ordering of specific treatment, testing, and investigative protocols by physicians. These tools may help reduce unwarranted clinical variation and improve health care efficiency. Despite their routine implementation within electronic medical records (EMRs), little is understood about how they are used and what factors influence their adoption in practice.

**Objective:**

This study aims to (1) describe the patterns of use of standard order sets implemented in a widely used EMR (PowerPlans and Cerner Millennium) within a multihospital digital health care system; (2) explore the experiences and perceptions of implementers and users regarding the factors contributing to the use of these standard order sets; and (3) map these findings to the Capability, Opportunity, and Motivation Behavior (COM-B) model of behavior change to assist those planning to develop, improve, implement, and iterate the use of standard order sets in hospital settings.

**Methods:**

Quantitative data on standard order set usage were captured from 5 hospitals over 5-month intervals for 3 years (2019, 2020, and 2021). Qualitative data, comprising unstructured and semistructured interviews (n=15), were collected and analyzed using a reflexive thematic approach. Interview themes were then mapped to a theory-informed model of behavior change (COM-B) to identify determinants of standard order set usage in routine clinical practice. The COM-B model is an evidence-based, multicomponent framework that posits that human actions result from multiple contextual influences, which can be categorized across 3 dimensions: capability, opportunity, and motivation, all of which intersect.

**Results:**

The total count of standard order set usage across the health system during the 2019 observation period was 267,253, increasing to 293,950 in 2020 and 335,066 in 2021. There was a notable shift toward using specialty order sets that received upgrades during the study period. Four emergent themes related to order set use were derived from clinician interviews: (1) Knowledge and Skills; (2) Perceptions; (3) Technical Dependencies; and (4) Unintended Consequences, all of which were mapped to the COM-B model. Findings indicate a user preference for customized order sets that respond to local context and user experience.

**Conclusions:**

The study findings suggest that ongoing investment in the development and functionality of specialty order sets has the potential to enhance usage as these sets continue to be customized in response to local context and user experience. Sustained and continuous uptake of appropriate Computerized Provider Order Entry use may require implementation strategies that address the capability, opportunity, and motivational influencers of behavior.

## Introduction

Electronic medical records (EMRs) are widely used in hospital settings worldwide, particularly in high-income countries, to improve efficiency by streamlining data access, reducing paperwork, and enhancing clinical workflows [1-3]. Information captured in EMRs is utilized by computerized clinical decision support systems to provide timely assistance to clinicians at the point of care, facilitating high-quality, high-value care. An important mechanism by which EMRs help reduce unwarranted clinical variation is through clinical decision support via the use of standard order sets [4]. Standard order sets allow care providers, most commonly doctors, to order or prescribe a standardized list of care elements (eg, pathology, imaging, and medication requests) that have been identified as evidence-based care for specific conditions or clinical pathways [5].

Traditionally, these standard order sets were implemented as standalone Computerized Provider Order Entry (CPOE) systems. However, with the widespread adoption of EMRs, they are now integrated as a module or subsystem within them [4,5]. Standard order sets within EMRs enable clinical teams to electronically order the necessary care elements, with networked communication delivering the order directly to the provider responsible for fulfilling it. Consequently, these electronic order sets, such as other types of digital health interventions, are only as effective as their appropriate uptake by clinical end users [6]. Ensuring this uptake requires a range of implementation and human factor strategies, including (but not limited to) integration with clinical workflows [7,8], training and awareness of the technology [9], end user perceptions of benefit [10], planned adoption strategies [11], and adequate resourcing [12].

Therefore, setting up standard order sets and maintaining them over time within proprietary EMRs can be resource-intensive for health care organizations. It often requires input from a range of clinical and nonclinical stakeholders to ensure that order sets and related workflows are appropriate, safe, effective, and acceptable to end users [13,14]. Multiple iterations and upgrades are necessary to maintain their purpose, stay aligned with the latest guidelines, and improve functionality and user experience, all with the aim of optimizing use and clinical effectiveness [15].

Despite their widespread use in hospitals with EMR systems, the evidence base for the effectiveness of standardized order sets in reducing unwarranted variation and promoting high-value care is not yet well established [16]. Furthermore, a gold-standard approach for managing order set implementations in hospital EMRs throughout their life cycle to optimize effectiveness in reducing unwarranted clinical variation has yet to be established. A recent systematic review examined the evidence for 2 widely used EMR systems that implement standard order sets to reduce clinical variation: PowerPlans (Cerner) and SmartSets (Epic). Of the 36 included studies, most (n=30) reported favorable findings, but only half (n=13) measured the impact on clinical variation. The authors of the review concluded that high-quality study designs were rare [15]. Numerous patient-, clinical-, and organization-related factors likely influence the effectiveness, or lack thereof, of standard order sets implemented in hospital EMRs [14,15,17]. A key factor that underpins the potential impact of these order sets on patient care is whether doctors in EMR-enabled hospitals actually use the order sets available to them [7,18].

Although it is possible to enforce the use of specific standard order sets by embedding forcing functions in EMRs, doctors in hospital settings typically have the discretion to decide whether and when to initiate a standard order set. Consequently, they may or may not use these order sets frequently in their day-to-day practice. Additionally, the implementation of electronic standard order sets is often dynamic; as new sets are introduced, existing sets are updated based on user feedback or contextual changes, and some are removed from EMR systems [19]. Patterns of standard order set use in hospitals with integrated EMRs, both over time and across specialty areas, remain a relatively unexplored area of research [20]. However, this topic has the potential to provide valuable insights for those seeking to reduce unwarranted variation through the optimal use of standard order sets to promote high-value care and reduce low-value care [21].

Using order sets in routine clinical practice is likely to be influenced by multiple intersecting behavioral factors, which, to our knowledge, have not been contextually identified in the published literature [22]. One way to qualitatively taxonomize user-perceived influences associated with CPOE use is to map user experiences and perceptions to the Capability, Opportunity, and Motivation Behavior (COM-B) model of behavior change [23,24]. The COM-B model is an evidence-based, multicomponent framework of behavior that posits human actions result from various contextual influences, which can be categorized across 3 dimensions: capability, opportunity, and motivation. These dimensions intersect with one another [25]. This model has been effectively utilized to identify, predict, and, where necessary, change behaviors across various health settings, but it has not yet been frequently applied within the context of digital health [23,24,26,27].

This study aimed to (1) describe the patterns of use of standard order sets implemented in a widely used EMR (PowerPlans [PowerPlan, Inc./Roper Technologies] and Cerner Millennium [Oracle Health]) within a multihospital digital health care system; (2) explore the experiences and perceptions of implementers and users regarding the factors contributing to the use of these standard order sets, and map these findings to the COM-B model to assist those planning to develop, improve, implement, and iterate the use of standard order sets in hospital settings.

## Methods

### Setting and Study Context

This study was conducted in a large public health system comprising 5 hospitals with varying characteristics, serving a broad geographical catchment area of 3856 km^2^ in Australia. The largest referral center was an inner metropolitan teaching hospital (“Hospital 1,” with 1033 beds), offering all major medical and surgical specialties except for maternity services and pediatrics. The second largest hospital in the study (“Hospital 2,” with 459 beds) was also located in a metropolitan center and included most medical and surgical specialties, including maternity and pediatric services. The third (“Hospital 3,” with 239 beds) and fourth (“Hospital 4,” with 199 beds) largest hospitals were also situated in metropolitan areas but offered a smaller range of surgical and nonsurgical specialties. The smallest hospital (“Hospital 5,” with 29 beds) was in a rural town and provided some specialty services, including maternity care.

Each of the 5 hospitals demonstrated a relatively high level of digital maturity that predated the commencement of this study. They were using the same instance of an integrated EMR (Cerner Millennium), which was implemented iteratively across the study sites from 2015 to 2018. Standard order sets were integrated into this EMR through a proprietary CPOE subsystem known as “PowerPlans.” Within this EMR system, standard order sets are accessed through a clickable icon, and each standard order set comprises a collection of grouped orders. These order sets also include functionality that allows for the incorporation of written clinical decision support or hyperlinks to relevant supporting evidence, if needed. The orders are diverse in nature, encompassing specific patient care tasks; care plans with identified clinical or functional outcomes; investigations, including imaging and pathology; medications, fluid orders, blood products, and nutritional supplements; and requests for consultations with other clinicians. Each standard order set comprises a collection developed through clinician-led initiatives, with broad stakeholder engagement and approval from relevant clinical networks and internal hospital governance processes before implementation. Each standard order set available in the EMR is categorized into a specific clinical specialty area when applicable; otherwise, it is classified as “other” if the order set is generic in nature and available for use by all clinicians or cannot be classified into a specialty area. This specialty area classification was carried out by representatives from the health system’s digital health and informatics clinical liaison service, who are familiar with the development, iteration, maintenance, and clinical governance of standard order sets. The development and iteration of specialty-based standard order sets are typically clinician-led, with broad stakeholder consultation before implementation. In addition to routine system maintenance and updates, as well as minor iterations of individual standard order sets, a major system-wide review and update for medication management and anesthetics-related standard order sets was conducted in this EMR before the 2020 data extraction.

### Ethical Considerations

Ethics approval was granted by the Metro South Human Research Ethics Committee for ethical and scientific review (HREC/2020/QMS/64807). Participation was voluntary, and participants could withdraw at any time. All data were anonymized or deidentified and handled in accordance with the guidelines of the Metro South Human Research Ethics Committee.

### Study Design

This mixed methods design included quantitative data on standard order set usage captured over 5-month intervals (January to May) for each of the 3 years (2019, 2020, and 2021). It also involved a qualitative context assessment with relevant stakeholders to examine the reasons behind usage patterns by exploring doctors’ experiences and perceptions of the factors contributing to the use of these standard order sets.

### Quantitative Data Collection

To address the first study aim, usage data for all standard order sets was extracted from the EMR for each of the 5 participating hospitals for the entire months of January to May in 2019, 2020, and 2021. Experts, who are key stakeholders in the implementation of standard order sets, recommended this specific time frame for meaningful comparisons to account for disruptions in the latter half of these years due to EMR upgrades. The extracted data included dates, the types of standard order sets used, and the location (hospital) of use, but did not include individual patient identity information.

### Qualitative Data Collection

Members of the digital adoption team (implementers; n=6) were invited to participate in interviews as part of the initial context assessment to understand how standard order sets were adopted and upgraded across the health system of interest. Participation was voluntary and conducted during work hours, either in person or via Microsoft Teams (Microsoft Corp.) videoconferencing. Participants could withdraw without penalty at any time. The context assessment discussions lasted approximately 60 minutes and were audio-recorded with informed consent obtained before the interview.

Face-to-face semistructured interviews targeting registered medical officers were also conducted. It is important to note that nurses and student doctors were not eligible for this study, as only registered medical officers are legally permitted to order or modify medications and medical procedures in Queensland, which is the primary purpose of “PowerPlans.” Purposive sampling for maximum variation in clinical seniority and inclusiveness of representatives from specialties with low, medium, and high use of standard order sets was conducted through email invitations (n=15) facilitated by health service staff familiar with this study. Participation was voluntary and conducted during work hours, either in person or via Microsoft Teams videoconferencing. Participants could withdraw without penalty at any time. Participants were prompted to share their experiences and broader perceptions of using standard order sets in their routine clinical practice. The semistructured interviews explored various topics, including experiences and perceptions regarding the types of standard order sets used, reasons for their use, perceived impacts on workflow and clinical outcomes, and opinions on standard order sets as clinical decision support tools. Each interview lasted approximately 45 minutes and was audio-recorded, with informed consent obtained before the start of the session.

### Reflexivity and Rigor

Semistructured qualitative interviews were conducted by a researcher (SN) experienced in qualitative research, including semistructured interviews with medical professionals in health care settings. The interviewer was not previously known to, nor in a dependent relationship with, the potential participants and had no prior personal experience using standard order sets. This approach minimized the potential for bias that could influence the interview process and data collection. The trained researcher (SN), an experienced health systems scientist, was equipped to effectively navigate and explore the complexities of the subject matter while being mindful of the differing dynamics between participant and interviewer. The interviewer engaged in discussions with the research team and created memos and field notes to practice critically conscious reflection and to remain aware of their role in the collection, analysis, and interpretation of the interview data [28]. Additional rigor was ensured through member checking during face-to-face interviews, where participant statements were restated for clarification and understanding [29].

### Data Analysis

Overall standard order set usage was aggregated by hospital facility and specialty area for each of the 3 data collection periods. Alluvial plots were used to visualize trends in usage over time within each specialty order set area across the 3-year data capture periods in each hospital. Two series of plots were prepared: (1) standard order set usage expressed as raw totals for each hospital; and (2) standard order set usage expressed as a percentage of total standard order set use for each hospital. Additionally, the 5 most used standard order sets were tabulated, including a brief description of each order set’s purpose.

Analysis of the qualitative data was conducted iteratively, with modifications made to the interview guide questions and probing after the first 2 interviews. This aimed to capture the relevance of standard order sets to the clinical roles of health professionals and to explore the reasons behind usage patterns. Audio recordings were transcribed verbatim, and the interviews were rapidly analyzed using an inductive reflexive thematic approach to identify emergent themes [30]. This involved analyzing the transcripts thematically in parallel with data collection to identify data saturation. One researcher (SN) independently read through the interview transcripts to identify codes, which were subsequently discussed with 2 other researchers (SM and HM) separately. This iterative process led to the generation and grouping of code categories, resulting in the creation of distinct themes. Consensus on the final themes was achieved before mapping these findings to the COM-B model. Framework analysis was then utilized to deductively map the themes to the COM-B model of behavior change, thereby identifying the capability, opportunity, and motivational influences associated with the use of standard order sets in routine clinical practice.

## Results

### Patterns of Standard Order Usage

**Figure 1:**
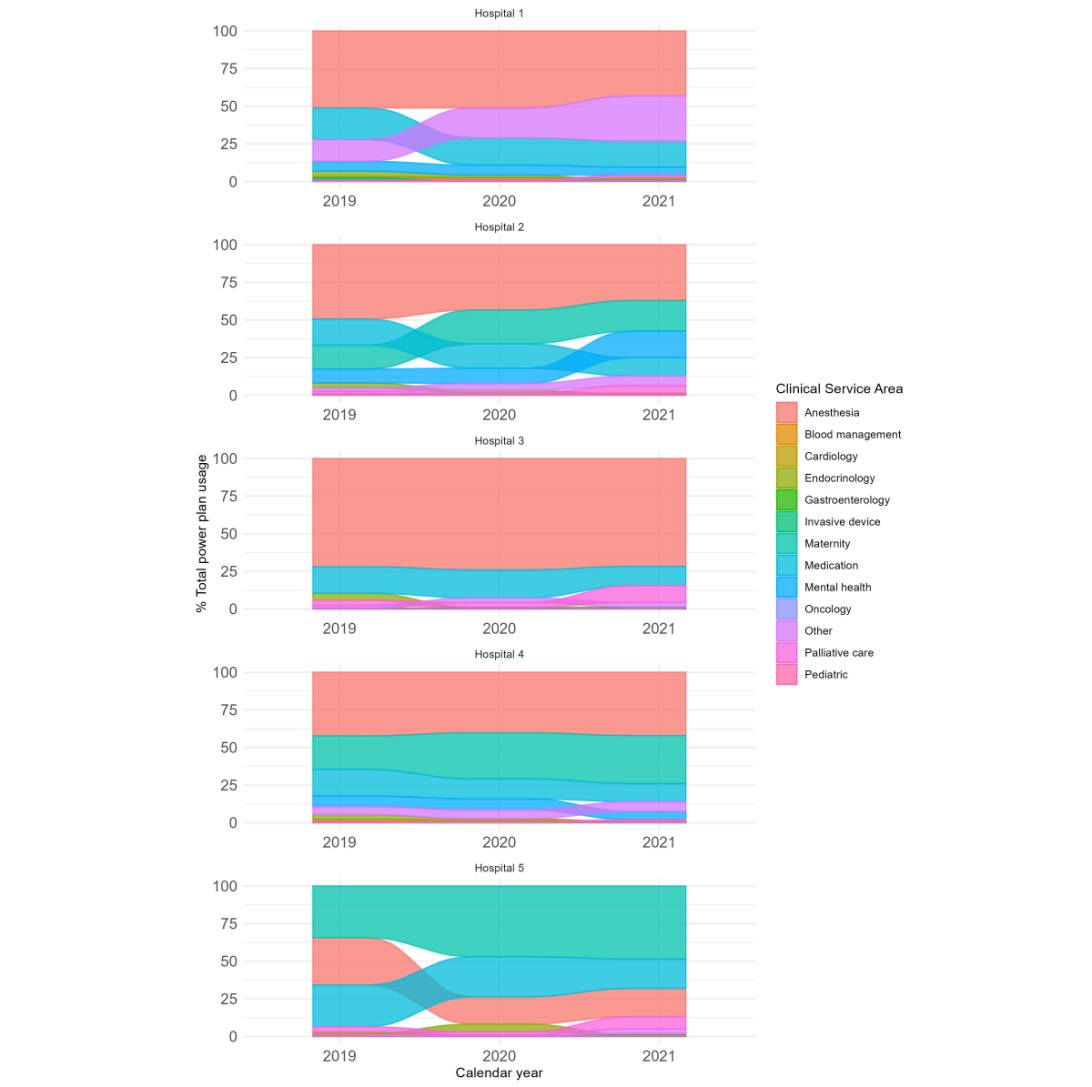
Total standard order set usage by clinical specialty area across each study period for each participating hospital.

The patterns of total order set usage by specialty areas and hospitals across the observation periods are illustrated in Figure 1. The total use of standard order sets across the health system during the 2019 observation period was 267,253. This figure increased to 293,950 in 2020 and 335,066 in 2021. Differences in total usage counts between hospitals corresponded with the relative sizes of the participating hospitals.

**Figure 2:**
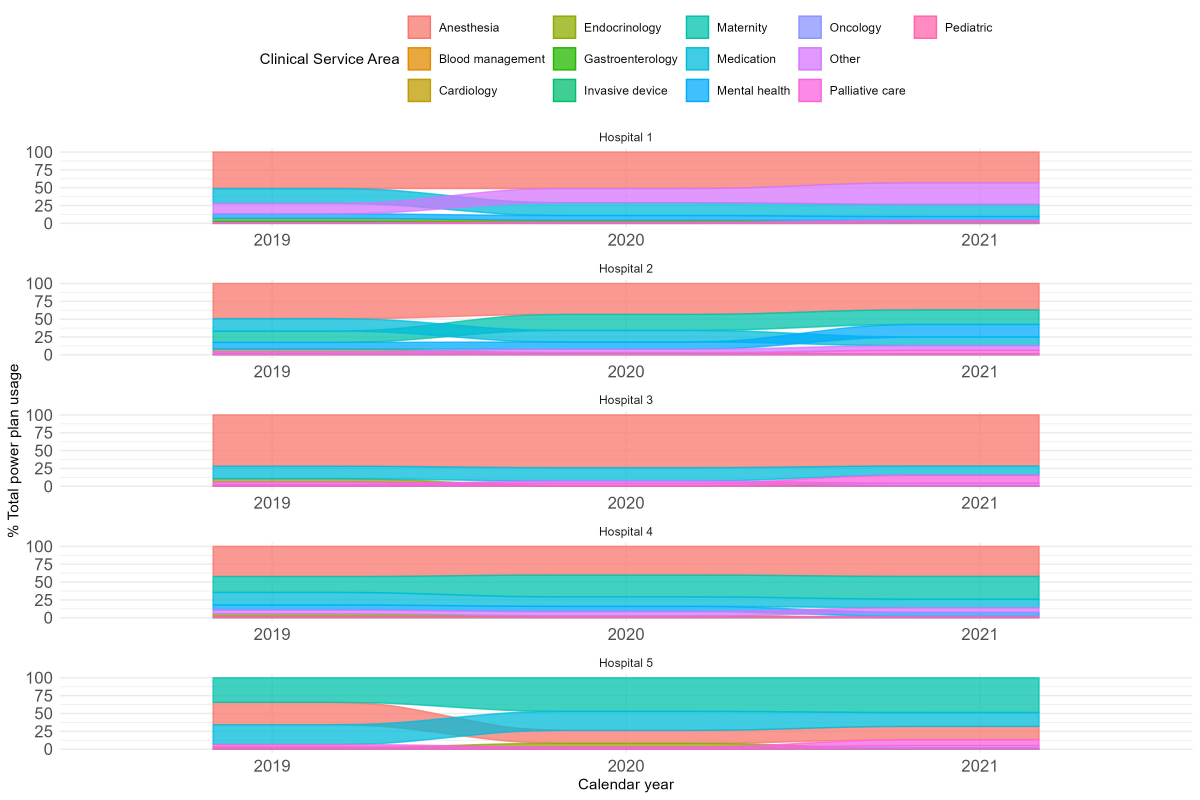
Percentage of standard order set usage by clinical specialty area across each study period for each participating hospital.

There were considerable differences among hospitals regarding the specialty areas of order sets. However, these differences reflected and aligned with the expected variance in case mix and activity levels between hospitals. For instance, the large university teaching hospital, which had a high case-mix activity, also exhibited high usage of anesthetic-related order sets. Similarly, hospitals with a higher proportion of maternity service activities in their case mix demonstrated greater utilization of maternity-related order sets. Specialty area order set usage, expressed as a percentage of total order set use per calendar year for each hospital, is presented in Figure 2. The use of specialty order sets evolved over the 3 years. There was an overall shift in the pattern of standard order set template usage, moving away from more generic sets that could not be classified into specific specialty clinical areas (categorized as “other”) toward those that were more clearly customized to the requirements of specialty areas of clinical practice. This trend indicates that the development and iteration of standard order sets available for use continued to influence usage patterns throughout the 3-year period.

Overall, the 5 most utilized order sets across the entire study period were Acute Pain Management (n=325,664), Acute Behavioural Disturbance Management Adult (Mental Health; n=63,354), VTE Prophylaxis (n=51,023), Healthy Newborn Protocols (Maternity) (n=26,229), and Postoperative Nausea and Vomiting (Anaesthesia; n=25,458). These highly utilized specialty order sets share the overarching characteristics of being high-volume, protocolized components of patient care delivered in hospital settings, where minimizing unwarranted clinical variation is likely to benefit both patients and the health service. The purpose of each of these highly utilized standard order sets is summarized in Table 1.

**Table 1:** Descriptions and total usage frequency of the 5 most widely used order sets across all participating hospitals for the overall study period.

Standard order sets	Overall use (frequency of patient encounters), n	Description
Acute Pain Management (Adult)	325,664	Used to order and chart scheduled analgesics, including opioids, barbiturates, and dissociative anesthetics. This plan serves multiple purposes, including fast-tracking orders for patients in severe acute pain or those undergoing surgical procedures. As a result, it is utilized in general and emergency medicine, anesthesia, and some palliative care settings.
Acute Behavioural Disturbance Management (Adult)	63,354	Initiating this power plan is recommended for new admissions to the acute psychiatry ward and includes a series of psychiatric behavioral assessments and testing protocols required before charting psychotropic medications.
VTE^a^ Prophylaxis	51,023	The VTE prophylaxis power plan includes a series of risk assessments and protocolized interventions designed to reduce the likelihood of VTE in at-risk patients after admission.
Healthy Newborn (Protocols)	26,229	The electronic adaptation of the newborn assessment protocol, mandated by the Queensland government, is required following an infant’s birth and within 48 hours before hospital discharge. This includes guidelines with reminders for physical examinations, which are considered the most important screening tool for major occult congenital anomalies.
Post-Operative Nausea and Vomiting (PONV)	25,458	Nausea and vomiting are common adverse events following anesthetic procedures, typically with a complex and multifactorial etiology. The initiation of this standard order set is recommended for all patients undergoing surgical procedures with general anesthesia at participating study hospitals. It includes an evidence-based PONV risk assessment for the administration of prophylactic monitoring and treatment.

^a^VTE: venous thromboembolism.

### Factors Influencing Standard Order Set Implementation and Use

All 6 members of the digital adoption team participated in a series of 3 unstructured discussions aimed at understanding the implementation context for standard order sets and the development process of these order sets. Additionally, 9 medical officers consented to and engaged in face-to-face interviews to explore the factors influencing clinicians’ usage of standard order sets. Discussions with the digital adoption team also informed the iteration of our face-to-face clinician interview questions, ensuring we had sufficient contextual knowledge to engage in meaningful conversations and obtain rich data. The participants included 3 junior medical officers, 3 registrars, and 3 consultants.

### Reflexive Thematic Mapping to the COM-B Model

#### Themes Overview

Four overarching emergent themes related to the use of standard order sets were derived from the qualitative data: (1) Knowledge and Skills; (2) Perceptions; (3) Technical Dependencies; and (4) Unintended Consequences. When considered within the context of the COM-B model, these emergent themes aligned with the Capability (Physical and Psychological), Opportunity (Physical and Social), and Motivation (Reflective and Automatic) influences on behavior, as mapped in Table 2.

**Table 2:** Emergent themes and subthemes mapped to the COM-B^a^ model.

COM-B component	COM-B influencer	Emergent theme	Subthemes
Capability	Psychological capability	Knowledge and Skills	(1) Education and Training and (2) Awareness of Standard Order Sets
Opportunity	Social opportunity	Perceptions	(4) Access to Support and (5) Culture
Opportunity	Physical opportunity	Technical Dependencies	(7) Interoperability, (8) Infrastructure Pipeline, and (9) Interface and Alert Fatigue
Motivation	Automatic (personality and emotions)	Knowledge and Skills	(3) Willingness to Learn
Motivation	Reflective (evaluations)	Unintended Consequences	(6) Clinical and Workflow Improvements, (10) Inappropriate Dosing, and (11) Medication Delay

^a^COM-B: Capability, Opportunity, and Motivation Behavior.

#### Knowledge and Skills (Psychological Capability and Automatic Motivation)

The theme of Knowledge and Skills encompasses the cognitive and motivational mental processes necessary to engage in a particular task or activity. This theme was mapped to 2 interrelated behavioral influences: psychological capability and automatic motivation. Psychological capability pertains to factors associated with learning, attention, and memory [31] and encompasses the following subthemes: (1) Education and Training, and (2) Awareness of Standard Order Sets. Automatic motivation encompasses the processes related to reactions, desires, and drive states [31], with a specific focus on the subtheme (3) Willingness to Learn. While there was some variation in experiences regarding Education and Training across clinical areas, these experiences could be broadly categorized into 2 overlapping groups: formal training and informal mentoring. For instance, interviewees from 2 clinical specialties noted that they received specialized training focused on a select group of frequently used standard order sets. As a result, these interviewees indicated that clinicians within their specialties were often not familiar with PowerPlans outside their specific areas of practice. Conversely, a respondent from a different medical specialty reported that training was less formal and primarily relied on experiential learning, supplemented by some informal mentoring. One interviewee mentioned that they often “phone a friend,” while another remarked that they “practice on the run.” Awareness of standard order sets as clinical decision support tools, while linked to education and training, emerged as a distinct factor that could mediate a user’s knowledge and skills. This awareness was not consistent across interviewees. For example, one participant viewed standard order sets as “straight translations of (former) paper-based ordering set forms,” whereas another described them as “knowledge translation tools.” Additionally, interest in developing proficiency or gaining a greater understanding of the suite of standard order sets available beyond their formal education and training varied markedly among respondents. This variation was classified under the subtheme of Willingness to Learn.

#### Perceptions (Social Opportunity and Reflective Motivation)

The theme of perceptions encompasses participants’ views regarding the intrapersonal, interpersonal, and environmental contexts that influence their thoughts about a particular activity or resource. This theme was mapped to the following behavioral influences: social opportunity and reflective motivation. Social opportunity pertains to the opportunities presented by interpersonal influences, social cues, and cultural norms [31]. It includes the subthemes of (4) Access to Support and (5) Culture. Reflective motivation refers to the processes involving intentions and evaluations, including beliefs about what is considered good or bad [31]. This theme encompasses the subtheme of (6) Clinical and Workflow Improvements. Participants expressed varying assessments of the technical and ongoing professional support available to them for using standard order sets. Those from 2 clinical specialties often relied on troubleshooting problems or making modifications to plans through consultation with their peers or line managers alone. This contrasted with participants from 3 other clinical specialties, who collaborated closely with digital support services beyond their specific areas to update and further modify standard order sets when appropriate. Upon further inquiry, differences in perceptions regarding the availability and quality of support outside their immediate specialties emerged. For instance, one participant expressed that they “have no awareness around support,” while another indicated that there is “consistent support” and a “bi-monthly working group.” These varied perceptions of organizational support intersected with participants’ views on organizational culture as it relates to digital health. One participant highlighted the “hierarchical nature of medicine,” which may discourage peers from seeking greater engagement with standard order sets beyond their immediate designated needs. By contrast, those who accessed organizational support described a “mature digital culture” and perceived ongoing support as readily available when needed. Overall, there appeared to be a consensus among interviewees that standard order sets positively impacted patient outcomes and enhanced their workflows. A senior clinician observed that the “risk of over-sedation, respiratory depression, and death has substantially improved” due to the use of standard order sets for charting the titration of psychotropics, such as clozapine. A peer from another specialty noted that “clinical handover improved.” Interviewees frequently described the use of standard order sets in routine practice with terms such as “timesaving” and “efficiency gain.” One participant remarked that it contributed to the team having a “common clinical language when we talk about patients.”

#### Technical Dependencies (Physical Opportunity)

Technical dependencies refer to the interconnections within the digital ecosystem that rely on one another to complete mutual tasks and activities. This concept is mapped to the behavioral influence known as physical opportunity, which describes the environmental context in which a task or activity occurs, as well as the physical resources required to perform it [31]. The subthemes include (7) Interoperability, (8) Infrastructure Pipeline, and (9) Interface and Alert Fatigue. Within the broader health service digital ecosystem, charting systems across specialty areas did not always integrate effectively, resulting in interoperability gaps for modules such as standard order sets. This issue was particularly evident in oncology, which utilized a separate ordering and charting system that was not interoperable with the broader EMR. One participant noted that chemotherapy prescriptions were dispensed and tracked separately. Interviewees expressed that even if there was a desire to enhance visibility and training for standard order sets—such as the adult palliative care standard order sets, which could assist “non-palliative care clinicians in caring for dying patients outside our unit,” this expansion was contingent upon “using the pipelines,” which required sociotechnical and peer support. One respondent noted that this “involves education, nursing support, and medical support. At this stage, we don’t have the capacity.” Alert fatigue was most frequently reported with the generalized standard order sets, particularly for venous thromboembolism prophylaxis and heparin infusion, which can “overcrowd the screen” and lead to the “accidental initiation of orders,” as mentioned by one interviewee. Several participants commented on the “old-fashioned” interface, suggesting it could “use a refresh,” thus highlighting an opportunity for the application of user-experience design principles.

#### Unintended Consequences (Reflective Motivation)

Unintended consequences refer to the unplanned “costs” associated with using or implementing a digital technology. These costs may be cognitive, social, or technical in nature. This theme was mapped as a motivational influencer [31], as it reflects how interviewees assessed the decision to use standard order sets in light of these unplanned costs across various contexts. Interviewees consistently identified 2 main subthemes regarding the unintended consequences of standard order sets use: (10) Inappropriate Dosing and (11) Medication Delay. There was concern that over-protocolizing medication titration could lead to inappropriate dosing for patients who fell outside standardized dosing norms. Notably, one interviewee remarked that “doctors may not check the maximum dose associated with a specific PowerPlan and may end up underdosing a patient.” This issue was illustrated with an example from their specialty area, where local (health district) guidelines establish a lower dosing threshold than the recommendations at the state level. In this case, the standard order sets’ titration phases defaulted to the local guideline unless overridden. Other interviewees echoed similar concerns about inappropriate dosing and the treatment of patients in general, with one describing it as “creating a culture of recipe shopping and inappropriate applications of certain prescriptions and investigations.” The issue of medication delay was also noted by interviewees as a separate unintended consequence of engaging with the standard order sets module. This delay was often related to accidental initiations or the discovery of mistakes after initiating a plan, which could hinder the approvals pipeline. This concern was particularly relevant when using the acute pain management standard order sets, designed to chart a range of scheduled analgesics. Such delays may contribute to user hesitancy, motivating physicians to “chart medications outside of standard order sets,” as one interviewee noted. Another interviewee explained that “if an order is canceled within a PowerPlan, it can’t be reordered on the same PowerPlan,” which forces them to initiate a new plan and rechart the order, inadvertently delaying the patient’s receipt of their medication.

## Discussion

Findings from this mixed methods study have highlighted the dynamic nature of electronic standard order set use within a large hospital system. While the differences in standard order set usage between hospitals were expected due to the varying characteristics of each institution, the changes in the frequency of standard order set use over the 3 study years were inconsistent across specialty areas and hospitals. The specialty areas that received more upgrades to standard order sets during the study period also experienced the largest growth in usage, particularly in the larger facilities where the case mixes associated with those order sets were greatest. This indicates that targeted investments aimed at increasing contextual customization, improving training, and enhancing the visibility of standard order sets may facilitate continued uptake and usage within and across relevant specialties.

Overall, the use of standardized order sets continued to mature over the study years. The trend shifted from generic order set templates to customized specialty area order sets, which were typically developed by clinicians and iterated after broad consultation with clinical stakeholders. This suggests that the digital hospital system is continuing to mature. For health service personnel and clinical teams who invest time and resources in the development, implementation, and iteration of specialty standard order sets via EMRs, the goal of reducing unwarranted clinical variation can only be achieved if these order sets are utilized in daily clinical practice. While this study could not establish causal links between increased use of specialty standard order sets and patient outcomes among those receiving care associated with these order sets, the evidence of higher utilization over time, as the digital hospital system continues to mature, is nonetheless an encouraging finding.

Findings from this study are consistent with and provide complementary information to previous research that has mapped the behavioral determinants associated with using multipronged or complex interventions within health systems [22,24,26,27]. User perceptions of factors related to standard order set use implemented through the standard order set CPOE system closely align with the COM-B model [23,32,33], which encompasses interactions among physical and psychological capability, reflective and automatic motivation, and physical and social opportunity. This underscores the complexity of fostering positive behaviors regarding standard order set use within large, complex adaptive hospital systems that encompass a variety of specialty areas in clinical practice. Such complexity suggests that the mere availability of technical solutions—specifically, the presence of standard order sets within an EMR—may be insufficient to effect behavioral change among clinicians and promote optimal use of those order sets. Effective implementation requires appropriate multifactorial plans [34]. This may involve implementation strategies such as education and training to enhance knowledge and practical skills; a persuasive communication approach that includes sharing positive outcomes; the application of rules and incentives along with the establishment of cultural expectations; ensuring that suitable physical and social contexts are provided; and reducing actual or perceived barriers to the use of standard order sets within digital hospital systems.

The triangulation of quantitative patterns in usage changes over time and findings related to user perceptions of associated factors also have important implications for those aiming to leverage ongoing improvements beyond the initial EMR implementations. Specifically, continuous system upgrades and enhancements in the years following initial EMR implementation have the potential to boost usage as order sets are further customized to local contexts. This underscores the likely need for continuous clinical and executive leadership, along with health service investment, to foster an organizational culture and provide resources that support the empowerment and engagement of clinicians. This approach is essential for optimizing the ongoing development, use, and iteration of standardized order sets in key practice areas. Additionally, this study highlighted the potential for utilizing standardized order set metadata to guide strategic engagement with clinical streams that may be underutilizing the benefits of these order sets in their practice.

A strength of this study was its mixed methods approach, which not only examined broader patterns of standard order set use but also investigated the factors influencing the utilization of this increasingly common care delivery intervention. Additionally, the inclusion of a range of hospitals and specialty areas over a longitudinal time series further enhanced the study’s robustness. However, the large-scale perspective involving multiple hospitals over several years also presented the limitation of relying solely on high-level quantitative data. This constraint restricted the ability to draw conclusions regarding the quality of completion or the patient impact of order completion, which lies beyond the scope of this study but remains a priority for future research. Similarly, it was not within the scope of this study to verify qualitative reports of user experiences beyond the descriptions provided by the interviewees. While physicians are the primary users of standard order sets in this specific health system, some nurses may be involved in implementing or recommending changes to these plans. Further research to understand the patterns of standard order set use by nurses, particularly in systems where their involvement is more commonplace, would likely provide valuable insights into the field.

Prospective research evaluating the patient impacts associated with initiatives to increase the use of standard order sets—potentially in combination with advanced decision support solutions—remains a priority for future studies.

## Abbreviations

COM-B: Capability, Opportunity, and Motivation Behavior
CPOE: Computerized Provider Order Entry
EMR: electronic medical record

## Data Availability

The data sets generated during or analyzed during this study are available from the corresponding author upon reasonable request.
